# DysPIA: A Novel Dysregulated Pathway Identification Analysis Method

**DOI:** 10.3389/fgene.2021.647653

**Published:** 2021-07-05

**Authors:** Limei Wang, Weixin Xie, Kongning Li, Zhenzhen Wang, Xia Li, Weixing Feng, Jin Li

**Affiliations:** ^1^College of Intelligent Systems Science and Engineering, Harbin Engineering University, Harbin, China; ^2^Key Laboratory of Tropical Translational Medicine, Ministry of Education, College of Biomedical Information and Engineering, Hainan Medical University, Haikou, China; ^3^College of Bioinformatics Science and Technology, Harbin Medical University, Harbin, China

**Keywords:** dysregulated pathway, enrichment analysis, differential co-expression, gene regulation, differential expression, differential variability

## Abstract

Differential co-expression-based pathway analysis is still limited and not widely used. In most current methods, the pathways were considered as gene sets, but the gene regulation relationships were not considered, and the computational speed was slow. In this article, we proposed a novel Dysregulated Pathway Identification Analysis (DysPIA) method to overcome these shortcomings. We adopted the idea of Correlation by Individual Level Product into analysis and performed a fast enrichment analysis. We constructed a combined gene-pair background which was much more sufficient than the background used in Edge Set Enrichment Analysis. In simulation study, DysPIA was able to identify the causal pathways with high AUC (0.9584 to 0.9896). In p53 mutation data, DysPIA obtained better performance than other methods. It obtained more potential dysregulated pathways that could be literature verified, and it ran much faster (∼1,700–8,000 times faster than other methods when 10,000 permutations). DysPIA was also applied to breast cancer relapse dataset and breast cancer subtype dataset. The results show that DysPIA is effective and has a great biological significance. R packages “DysPIA” and “DysPIAData” are constructed and freely available on R CRAN (https://cran.r-project.org/web/packages/DysPIA/index.html and https://cran.r-project.org/web/packages/DysPIAData/index.html), and on GitHub (https://github.com/lemonwang2020).

## Introduction

**FIGURE 1:**
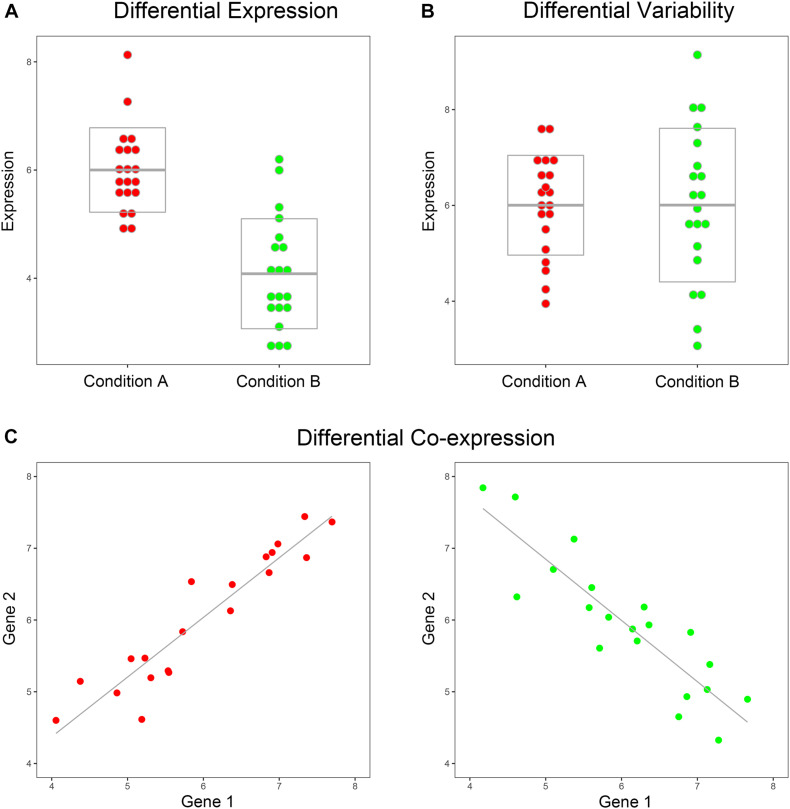
Toy example of DE, DV, and DC. (**A**) The means of gene expression are significantly different between the two conditions, such as case and control, while the variances are similar. (**B**) The variances of gene expression are significantly different between the two conditions, while the means are similar. (**C**) The correlation coefficients of the two genes are significantly different between the two conditions.

Over the past three decades, an amount of high-dimensional biological omics data types have emerged including genomics, sequencing, proteomics, epigenomics, and genome editing (Auffray et al., 2009). A common use of these data is to gather and compare samples from multiple conditions, e.g., disease and non-disease, cancer subtypes, drug sensitivity, and drug resistance, in an attempt to identify some biomarkers to distinguish between different conditions. Currently, the common methods of comparing samples from different conditions were Differential Expression (DE) analysis, Differential Variability (DV) analysis, and Differential Co-expression (DC) analysis (Ho et al., 2008; McKenzie et al., 2016) (Figure 1). In some research, DC gene pair was also called dysregulated gene pair. A range of statistical procedures, such as linear modeling (Smyth, 2004), SAM (Tusher et al., 2001), Bayesian methods (Hardcastle and Kelly, 2010; Hardcastle, 2016), and F test (Cui and Churchill, 2003), have been devised for accurate and efficient identification of DE and DV genes. Distinct from DE and DV, the DC methods that emerged to gain insights into the difference in gene–gene relationships between various conditions were gene-pair (regulation) centroid methods rather than individual-gene centroid methods. Currently, most of the DC methods relied on the Pearson correlation coefficient (PCC), such as DiffCorr (Fukushima, 2013), link-based DCEA (Yu et al., 2011), and DGCA (McKenzie et al., 2016). In biological pathways with gene interactions or regulations, DC gene pairs can also be considered as dysregulated gene pairs. The Correlation by Individual Level Product (CILP) was proposed in Lea et al. (2019) article to identify factors associated with interindividual variation in correlation. CILP can be used to estimate the dysregulated status of each gene pair between case and control samples. In the CILP method, there was a unique score for each gene pair in each sample, rather than a summary PCC statistic for a group.

Compared with gene-level analysis, pathway analysis can help in getting an insight into biological mechanisms, drug response, and disease states (Kanehisa et al., 2012). Classical pathway enrichment analysis methods, such as DAVID (Huang da et al., 2009a,b), were based on overrepresented statistical tests (such as Fisher’s exact test and hypergeometric test) to assess whether DE/DV genes were overrepresented in a predefined pathway. As the most popular one in the second-generation methods, Gene Set Enrichment Analysis (GSEA) (Subramanian et al., 2005) started with ranking all genes according to their DE levels, and then used the weighted Kolmogorov–Smirnov statistic to test whether genes from a prespecified pathway were significantly overrepresented toward the top or bottom of the ranked gene list. The similar strategies were used in gene set analysis (GSA) (Efron and Tibshirani, 2007), Parametric Analysis of Gene set Enrichment (PAGE) (Kim and Volsky, 2005), and Significance Analysis of Function and Expression (SAFE) (Barry et al., 2005). These conventional methods of pathway analysis focused on gene marginal effects in a pathway and ignored gene interactions that may contribute to a phenotype of interest. For two genes in a pathway, neither of them may have an effect on a phenotype of interest. However, when they were jointly considered, they may have a significant effect on the studied phenotype due to the gene–gene interaction. In the third generation of pathway analysis methods, such as Signaling Pathway Impact Analysis (SPIA) (Tarca et al., 2009) and PAthway Recognition Algorithm using Data Integration on Genomic Models (PARADIGM) (Vaske et al., 2010), the pathway topology was incorporated into the analysis. However, this gene regulation information (edge) was just used to adjust the gene (node) value in these methods.

Since the measures of these methods are mainly based on individual gene levels, they can be deemed as node (gene)-centric methods. Although these methods have made success in identifying significant biological pathways, the gene-pair relationships have not been fully considered. Obviously, the regulation relationships among genes were also the fundamental components of pathways, and their changes may play an important role in altering the activities of pathways (Liu et al., 2012). The DC-based pathway analysis aimed to identify pathways with more gene regulation differences related to the phenotype of interest. There were several studies focusing on DC-based pathway analysis. However, it is still not fully considered, and some improvements are needed.

Choi and Kendziorski proposed Gene Set Co-expression Analysis (GSCA) to identify differentially co-expressed (DC) gene sets (Choi and Kendziorski, 2009). Pairwise co-expressions were calculated for all the gene pairs within a pathway, then a dispersion index was introduced to quantify the differences between conditions, and samples were permuted across conditions to simulate the null distribution of equivalent correlation between conditions to identify significant DC pathways. The GSCA approach did not require genes to be highly correlated under at least one biological condition, but there were two weaknesses. The first was that the sample sizes of different conditions were not considered while just using the PCC. The second was that pathways were considered as gene sets, but the gene regulations were not considered. For each pathway, all the possible gene pairs were used to calculate the dispersion index.

Rahmatallah et al. (2014) proposed Gene Sets Net Correlations Analysis (GSNCA). It was a multivariate differential co-expression test which accounted for the complete correlation structure between genes. In GSNCA, weight factors related to the eigenvector of the correlation coefficient matrix under specific conditions were assigned to genes in proportion to the genes’ cross-correlations. Samples’ condition labels were permuted to estimate the significant level. The same as GSCA, a pathway was considered as a gene set and the gene regulation relationships were not considered in GSNCA. The correlation coefficient matrix for all the genes in a pathway was used to calculate the weight factor.

Zhang et al. (2009) proposed a gene interaction enrichment analysis method named “Interaction-based Gene Set Analysis” (IB-GSA). It incorporated knowledge of pathways to identify enriched gene interaction effects on a phenotype of interest. In IB-GSA, for each gene pair, the *t*-test was performed to compare the Pearson correlations between different conditions, and the t statistic was transformed to z score. Then the GSA-like “maxmean” statistic (Efron and Tibshirani, 2007) was adopted to calculate a pathway score that reflected the degree of gene interaction enrichment for the pathway. The “restandardization” permutation method was implied to determine the pathway significance. At last, the estimated significance level was adjusted to account for multiple-hypothesis testing through a standard Benjamini–Hochberg (BH) (Benjamini and Hochberg, 1995) FDR analysis. The same as GSCA and GSNCA, in IB-GSA, a pathway was considered as a gene set and the gene regulation relationships were not considered. The “maxmean” statistic was calculated based on all the possible gene pairs in the pathway.

Han et al. (2015) proposed a mutual information (MI)-based Edge Set Enrichment Analysis (ESEA) method to identify dysregulated pathways. In ESEA, a MI-based Edge Score was calculated for each regulated gene pair in pathways, then GSEA-based enrichment scores for pathways were calculated, the significant *P*-values were estimated based on permutation, and lastly the BH-FDR analysis was performed to adjust the estimated significance level. In ESEA, the background gene-pair set was not sufficient, the MI-based Edge Score was novel but has less directly biological meaning than the classic correlation coefficient, and the computational speed was extremely slow due to the large pathway gene-pair database.

In this article, we proposed a novel method called Dysregulated Pathway Identification Analysis (DysPIA) to overcome these shortcomings. A pathway is represented by the regulated gene pairs, but not just a set of genes, which is used in the traditional pathway analysis. We adopted the idea of CILP into analysis and performed a fast GSEA-like enrichment analysis. First, we calculated a Dysregulated Gene Pair Score (*DysGPS*) for each gene pair of interest, which was an individual-level-based statistic instead of population-level. Then, we calculated the Dysregulated Pathway Score (*DysPS*) based on a GSEA-like formula. Lastly, permutation-based significant *P*-values were estimated and the BH-FDR adjustment was performed. Compared with the previous methods, DysPIA provided a much larger and proper gene-pair background, fully employed all the sample information, and gained more significant pathways with biological meanings at a faster running speed.

The R package “DysPIA” has been constructed and is publicly available on R CRAN^1^, and the R dataset package “DysPIAData” including gene-pair background and pathway list is also publicly available on R CRAN^2^. They are also available on GitHub^3^.

## Materials and Methods

### Flowchart of DysPIA

**FIGURE 2:**
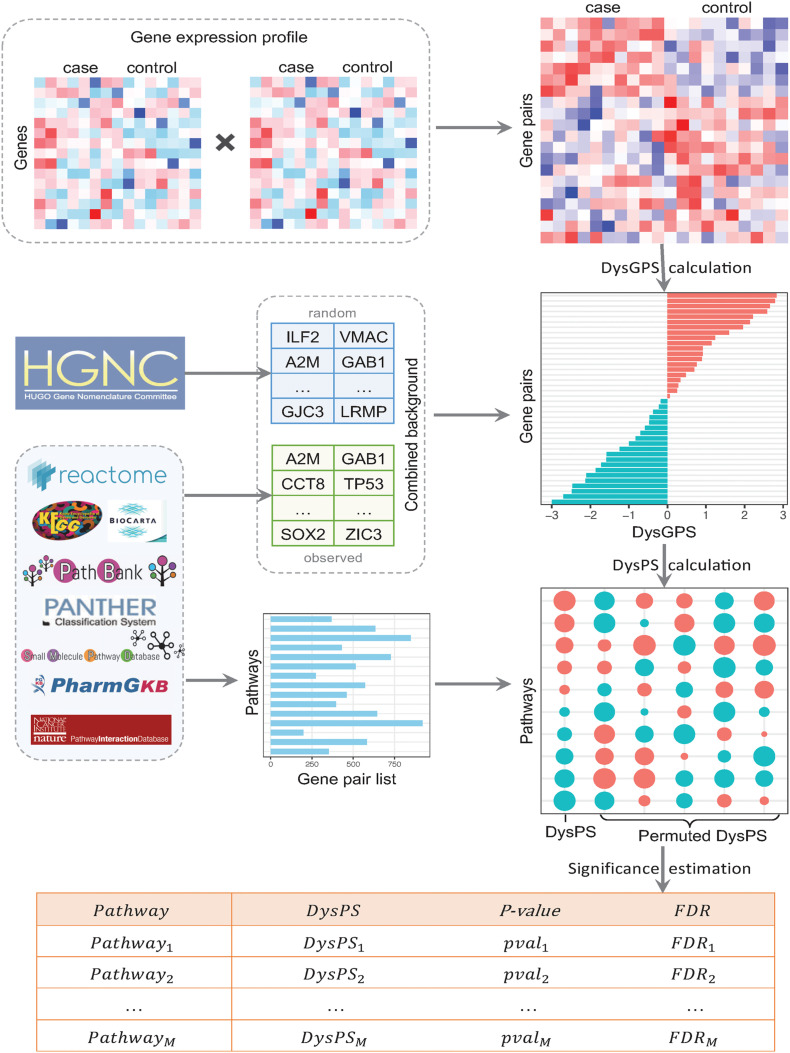
The flowchart of DysPIA. In the left side, there are the input data, gene expression, combined background, and pathway gene list. From the gene expression data, the CILP-like gene pair scores are calculated, then the *DysGPS* and *DysPS* are calculated in the right side. The results are shown in the bottom.

The overall procedure of DysPIA consists of two parts. The first part calculates the dysregulated score for each gene pair, and the second part calculates the dysregulated score for each pathway and estimates significance. The details are shown in Figure 2.

#### Part 1: Dysregulated Gene Pair Score (*DysGPS*) Calculation

Firstly, for each gene, the gene expression was mean centered and scaled separately in each group, e.g., case or control. Let *x* be the expression value of gene *X* across individuals in a population sample, with mean $\bar{x}$ and variance σ^2^. Standardization (*z*-score normalization) transforms were performed, such that the resulting distribution has a mean of 0 and a standard deviation of 1.

$$\tilde{x}=\frac{x-\bar{x}}{\sigma }$$

Secondly, for each gene-pair *X_i_* and *X_j_* from the combined-background set in sample *k*, the CILP-like statistic which was the product of these two genes’ standardized expression values was defined as:

$$y_{i,j}={\tilde{x}}_{i}^{k}{\tilde{x}}_{j}^{k}=\frac{\left(x_{i}^{k}-{\bar{x}}_{i}\right)\left(x_{j}^{k}-{\bar{x}}_{j}\right)}{\sqrt{\sigma_{i}^{2}\sigma_{j}^{2}}}$$

where, ${\tilde{x}}_{i}^{k}$ and ${\tilde{x}}_{j}^{k}$ are the standardized expression values for genes *X_i_* and *X_j_* in sample *k*, respectively.

Lastly, the dysregulated gene-pair score (*DysGPS*) was calculated based on a two-sample Welch’s *t*-test between groups:

$$DysGPS_{i,j}=\frac{{\bar{y}}_{i,j}^{case}-{\bar{y}}_{i,j}^{control}}{s_{\bar{△}}}$$

where, $s_{\bar{△}}=\sqrt{\frac{s_{1}^{2}}{n_{1}}+\frac{s_{2}^{2}}{n_{2}}}$ is the standard deviation of *y*_*i,j*_. Here $s_{i}^{2}$ is the unbiased estimator of the variance of *y*_*i,j*_ in each of the two groups with *n*_*i*_ = number of participants in group *L*.

#### Part 2: Dysregulated Pathway Score (*DysPS*) Calculation and Significance Estimation

A pre-ranked pathway enrichment analysis pipeline was utilized to calculate the *DysPS* and estimate the significance.

• Step 1: Rank the *N* gene pairs in the combined-background set in descending order based on *DysGPS* to form a gene-pair list *L* = {*gp*_1_, *gp*_2_, ⋯,*gp*_*N*_}.
• Step 2: Calculate *DysPS* for each pathway.

For a given pathway, gene pairs get different weights based on whether they are in the pathway. We evaluate the fraction of gene pairs in pathway *P(‘hit’)* weighted by their *DysGPS* and the fraction of gene pairs not in pathway *P(‘miss’)* present up to a given position *i* in *L*.

$$\begin{array}{c} S_{hit}\left(P,i\right)=\sum_{\begin{array}{c} gp_{j}\in P\\ j\leq i \end{array}}\frac{{\left|DysGPS_{j}\right|}^{p}}{N_{R}}\\ S_{miss}\left(P,i\right)=\sum_{\begin{array}{c} gp_{j}\notin P\\ j\leq i \end{array}}\frac{1}{N_{miss}} \end{array}$$

where, $N_{R}=\sum_{gp_{j}\in P}{\left|DysGPS_{j}\right|}^{p},\;N_{miss}$ represents the number of gene pairs in the list *L* and not in the pathway *P*.

$$\begin{array}{c} S_{max}=max\left(S_{hit}\left(P,i\right)-S_{miss}\left(P,i\right)\right)\\ S_{min}=min\left(S_{hit}\left(P,i\right)-S_{miss}\left(P,i\right)\right)\\ DysPS=\left\{ \begin{array}{c} S_{max},\left|S_{max}\right|>\left|S_{min}\right|\\ S_{min},\left|S_{max}\right|<\left|S_{min}\right| \end{array}\right. \end{array}$$

*DysPS* is the maximum deviation from zero of *S*_*hit*_ − *S*_*miss*_. For a randomly distributed *P*, the absolute value of *DysPS* will be relatively small, but if gene pairs in the pathway were concentrated at the top or bottom of the list, or otherwise nonrandomly distributed, then the absolute *DysPS* will be correspondingly high.

When *p* = 0, *DysPS* reduces to the standard Kolmogorov–Smirnov statistic; when *p* = 1, we are weighting the gene pair in the pathway by its absolute *DysGPS* normalized by the sum of the absolute *DysGPS* over all the gene pairs in the pathway. We set *p* = 1 for the examples in this article.

• Step 3: Randomly permute the sample labels, recalculate the *DysGPS* in the background, and recalculate the *DysPS* for each pathway.
• Step 4: Repeat step 3 for *n* times (*n* > 1000 is recommended) and create a distribution of the correspondingDysPS_NULL_.
• Step 5: Estimate the nominal *P*-value for *S* from DysPS_NULL_ by using the positive or negative portion of the distribution corresponding to the sign of the observed *DysPS*.
• Step 6: Estimate the false discovery rate (*FDR*) using the BH method.
  In the results, a pathway was said to have a gain of correlation if the *FDR* was significant and the *DysPS* value was positive, and a loss of correlation if the *FDR* was significant and the *DysPS* value was negative.
  Due to hundreds of thousands of gene pairs and thousands of pathways, a fast-calculation algorithm proposed in Korotkevich et al. (2019) article was utilized to speed up the analysis.

The R package “DysPIA” was developed and is publicly available on R CRAN.

### Simulation Data

To assess the power and performance of DysPIA, we performed several extensive simulations under various conditions. A total of 100,000 gene pairs were generated as background, and 1,000 pathways were generated in each simulation. For each gene pair, 100 case and 100 control samples were generated using the R package “MASS.”

Firstly, the gene-pair expression data was generated. A Bivariate Normal Distribution was supposed for each gene-pair expression.

$${\left(g_{i},g_{j}\right)}^{\prime }∼BN\left({\left(0,0\right)}^{\prime },\Sigma \right)$$

where, $\text{Σ}=\left(\begin{array}{cc} \text{1} & r_{k}\\ r_{k} & \text{1} \end{array}\right)$, *k* = 1, 2 for conditions 1 and 2.

In 100,000 gene pairs, 95,000 (95%) were set to be non-dysregulated and 5,000 (5%) were set to be dysregulated. In non-dysregulated gene pairs, the correlation coefficients for both conditions were set to be equal (*r*_1_ = *r*_2_) and they were randomly drawn from a uniform distribution between -1 and 1. In dysregulated gene pairs, five gradient correlation coefficient differences (0.4–0.8) between *r*_*1*_ and *r*_*2*_ were selected. In total, there were 10 groups of gain/loss of correlation dysregulated gene pairs with different degrees, which made them as much similar as we can to the real dysregulated gene pairs. The detailed gene-pair generations are shown in Table 1.

**TABLE 1:** The simulation data with 100,000 gene pairs.

Gene pair	Number of gene pairs		Correlation coefficients
		500	*r*_1_∼*U*[−0.6,1],*r*_2_ = *r*_1_−0.4
		500	*r*_1_∼*U*[−0.5,1],*r*_2_ = *r*_1_−0.5
GCDG	2,500	500	*r*_1_∼*U*[−0.4,1],*r*_2_ = *r*_1_−0.6
		500	*r*_1_∼*U*[−0.3,1],*r*_2_ = *r*_1_−0.7
		500	*r*_1_∼*U*[−0.2,1],*r*_2_ = *r*_1_−0.8
NDG	95,000		*r*_1_ = *r*_2_∼*U*[−1,1]
		500	*r*_1_∼*U*[−1,0.6],*r*_2_ = *r*_1_ + 0.4
		500	*r*_1_∼*U*[−1,0.5],*r*_2_ = *r*_1_ + 0.5
LCDG	2,500	500	*r*_1_∼*U*[−1,0.4],*r*_2_ = *r*_1_ + 0.6
		500	*r*_1_∼*U*[−1,0.3],*r*_2_ = *r*_1_ + 0.7
		500	*r*_1_∼*U*[−1,0.2],*r*_2_ = *r*_1_ + 0.8

*GCDG, gain of correlation dysregulated gene pair; NDG, non-dysregulated gene pair; LCDG, loss of correlation dysregulated gene pair.*

After the gene expression profiles of each gene pair *i* and *j* were generated, their product was set to be the gene-pair CILP score, and then the T-test-based *DysGPS* was calculated.

In the pathway generations, two parameters, the proportion of dysregulated gene pairs (*p*_dysgp_) and the pathway size (*n_p_*, number of gene pairs in a pathway), were designed. The five simulations with different proportions (20–60%) of dysregulated gene pairs were selected and compared. In each simulation, five different pathway sizes (20, 40, 60, 80, and 100) were selected, and 200 pathways were generated with each pathway size, respectively. Strong/weak dysregulated pathways and gain/loss of correlation dysregulated pathways were designed based on different parameters. In total, for each simulation, 1,000 pathways were generated, and 100 of them were dysregulated. The detailed pathway generations with a selected pathway size are shown in Supplementary Table 1.

In each condition, the Receiver-Operating Characteristic (ROC) curves were drawn based on sensitivity and specificity. Then the ROC analysis was used to evaluate and compare the performance of the algorithm under various scenarios.

### Gene Expression Dataset

Three gene expression datasets were used to evaluate the proposed method.

The first dataset was the p53 mutation data which detected gene expression in response to the status of transcription factor p53 in 50 NCI-60 cell lines with 17 cell lines carrying the native p53 status and 33 cell lines carrying the mutated p53 status (Olivier et al., 2002). This expression dataset for 10,100 genes was downloaded from the GSEA website, and 6,835 genes were used in the following analysis after matching the gene names with HGNC official gene symbols (Braschi et al., 2019). This dataset was used to compare our proposed method with other methods.

The second dataset was the breast cancer relapse dataset (GSE2034) (Wang et al., 2005) downloaded from NCBI Gene Expression Omnibus. There were 14,208 gene expressions in 286 lymph-node negative breast cancer patients including 179 relapse-free patients (controls) and 107 distant metastasis patients (cases). The dataset was divided into two groups based on ER status. There were 209 samples in the “ER+” group, including 129 control samples and 80 case samples. There were 77 samples in the “ER−” group, including 50 control samples and 27 case samples. The results for ER+ and ER− were compared and literature verified.

The third dataset was an RNA-seq expression dataset for breast cancer patients with different subtypes in The Cancer Genome Atlas (TCGA) (Vasaikar et al., 2018). It contained 20,155 genes in 1,041 breast cancer patients (40 normal breast samples were excluded). There were four breast cancer subtypes (Luminal-A, Luminal-B, Basal-like, HER2-enriched) based on the PAM50 model (Cancer Genome Atlas Network, 2012). There were 560 Luminal-A samples, 209 Luminal-B samples, 190 Basal-like samples, and 82 HER2-enriched samples. Dysregulated pathways between subtypes were identified using DysPIA.

### Background Gene-Pair Set and Pathway List

There were 19,297 genes in HGNC (Braschi et al., 2019) (version 01/31/2020), and it was impractical to use all possible gene pairs (>372 million) to be the background dataset. Therefore, a representative subset was critical in the pathway analysis. A combined background was proposed in DysPIA, including two parts. The first part was the retrieved gene pairs in the pathways from public pathway databases, and the second part was the randomly selected gene pairs.

For each pathway, only the regulated gene pairs are considered. It is significantly different to GSCA, GSNCA, and IB-GSA which simply include all n(n-1)/2 gene pairs. Based on the R package “graphite” (Sales et al., 2012, 2019), direct gene–gene regulations and metabolite-based propagated gene–gene regulations were retrieved from the pathways in eight public pathway databases for Homo Sapiens (version 01/31/2020), which are Reactome (Matthews et al., 2009), KEGG (Ogata et al., 1999), BioCarta (Nishimura, 2001), Panther (Mi et al., 2013), PathBank (Wishart et al., 2020), NCI/Nature Pathway Interaction Database (Schaefer et al., 2009), SMPDB (Jewison et al., 2014), and PharmGKB (Whirl-Carrillo et al., 2012). Totally, there were 333,484 gene pairs in 99,984 pathways. The details are shown in Table 2.

**TABLE 2:** Summary of pathways and gene pairs.

Database name	Number of pathways	Number of gene pairs
Reactome	1,901	264,867
KEGG	306	60,571
BioCarta	247	5,421
Panther	84	12,951
PathBank	48,593	6,882
NCI	212	14,198
SMPDB	48,581	6,777
PharmGKB	60	2,727
Total pathways	99,984	333,484
Random background	NA	349,915
Combined background	NA	682,417

These 333,484 gene pairs formed the first part of the background set, which was called the observed background gene-pair set. To avoid redundancy and easily query gene pairs in the database, in each gene pair, the genes were arranged in ascending order based on gene symbols, i.e., the first gene was smaller than the second one.

A total of 700,000 gene pairs were randomly selected from 19,297 genes in HGNC, and 349,915 of them in ascending order (about half of the selected gene pairs) remained as the random background gene-pair set. There were 982 gene pairs in common between the observed background and random background. Finally, their combination with 682,417 gene pairs was set to be the final background, which was called the combined background gene-pair set. In this combined background, there were similar numbers of gene pairs in the observed background and random background. The evaluation of the combined background is shown in section “Gene-Pair Background Evaluation.”

In total, there were 99,984 pathways retrieved which can be used in DysPIA. The KEGG pathways were used as examples in the following analysis.

## Results

### Simulation

**FIGURE 3:**
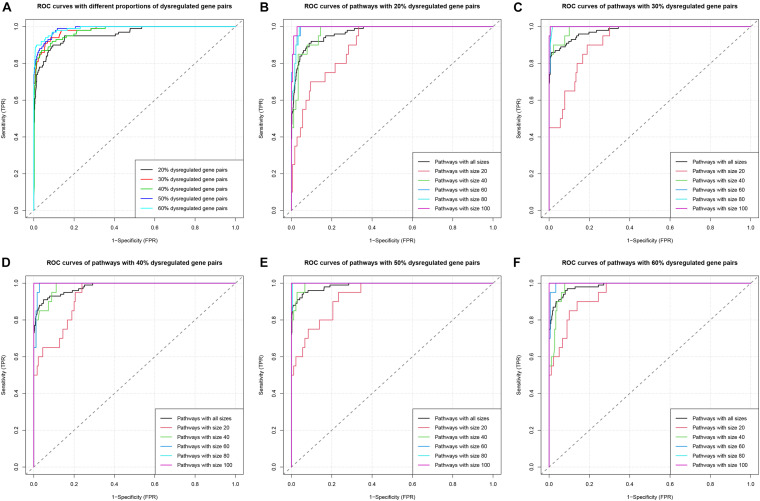
ROC curves in simulation study. (**A**) ROC curves in the five simulations with different proportions (20–60%) of dysregulated gene pairs. (**B**) ROC curves of pathways with different sizes (20, 40, 60, 80, and 100) when the proportion of dysregulated gene pairs is 20%. The group “Pathways with all sizes” means the union set of pathways with different sizes (20, 40, 60, 80, and 100). (**C–F**) Similar to **(B)**, ROC curves of pathways when the proportions of dysregulated gene pairs are 30–60%, respectively.

The Receiver-Operating Characteristic (ROC) analysis was used to evaluate and compare the performance of the algorithm under various scenarios (Figure 3). The area under the ROC curve (AUC) was calculated as the measure for comparison. Firstly, we compared the ROC and AUC among groups with different *p*_dysgp_ (Figure 3A). The overall AUC ranged from 0.9584 to 0.9896 and the AUC increased when the *p*_dysgp_ increased. Then within each *p*_dysgp_ group, the subgroups of different *n_p_* were compared (Figures 3B–F). The AUC increased when the pathway size increased for all the subgroups, and the AUC reached nearly 1 when there were 80 or more gene pairs in the pathway.

This simulation study indicated that DysPIA was able to identify the causal pathways with strong sensitivity and specificity.

### Gene-Pair Background Evaluation

The gene-pair background was evaluated based on p53 data. *DysGPS* was calculated in the observed background and random background separately, then the absolute scores were compared between these two groups using the Student T-test. The results showed that there were significant differences between them (mean absolute scores: 0.8150 vs. 0.8395, *P*-value: 2.14e-12). Therefore, the observed background was not good enough to be representative of the whole background, and the combined background was more real and critical.

Then we evaluated the sufficiency and the robustness of the sampling procedure since it was only about 1‰ of the whole gene pairs. We repeated the sampling procedure 20 times (randomly selected 700,000 gene pairs 10 times and 7,000,000 gene pairs 10 times). Then, *DysGPS* was calculated, and the absolute scores were compared between these groups using the Student T-test. The results showed that there were significant differences between the observed background group and each random background group (all *P*-values < 1e-10); no significant difference between the random background groups with 700,000 gene pairs and the random background groups with 7,000,000 gene pairs; and only 7 significant differences among the random background groups with 7,000,000 gene pairs under the threshold *P*-value < 0.05. The proportion of significant results is lower than the significance level (7/190 = 0.0368 < 0.05). Based on these results, we would say that this sampling procedure is robust. The detailed results are shown in Supplementary Table 2.

To evaluate the sufficiency of this combined background, we performed DysPIA using the whole background and compared it with the result using the proposed combined background. Based on the whole background, it took about 10 h to get the result with only 1,000 permutations, and we did not get the result with 10,000 permutations due to the large computational load, which indicated that it was impractical considering the whole background. The results using 1,000 permutations showed high consistency between the whole background and the combined background. The Pearson/Spearman correlation coefficient between them was 0.9973/0.9974. Sixteen pathways were significantly enriched under the threshold *P*-value < 0.01, and all of them were in the significant result based on combined background (17 pathways). The only pathway which was significant in the result based on combined background but not in whole background was “Cellular senescence.” Its *P*-value was 0.0112 which was close to the significant level. The detailed results are shown in Supplementary Table 3.

Based on these results, we would say that using a limited set of random gene pairs is as good as using the whole background.

### Computational Speed and Consistency

Since there was no public resource for IB-GSA, we compared DysPIA with ESEA, GSCA, and GSNCA.

**FIGURE 4:**
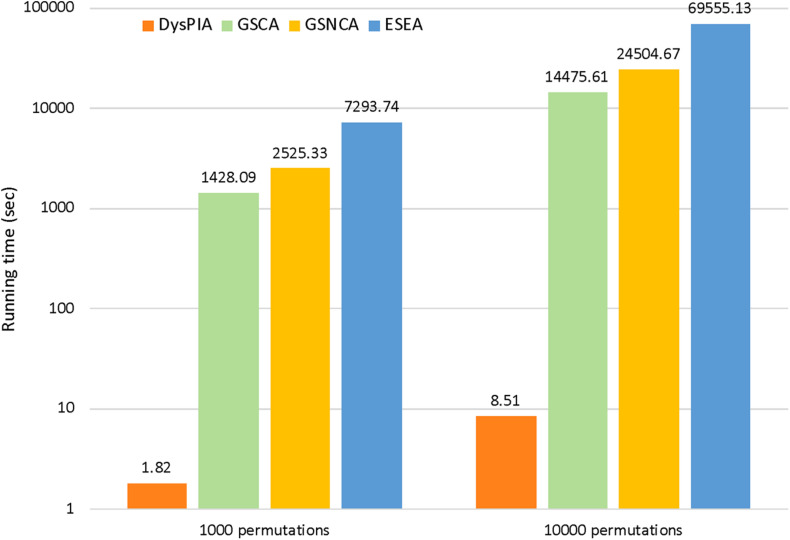
The running time comparison of the four methods.

In DysPIA, the idea of fast calculation proposed in FGSEA (Korotkevich et al., 2019) was applied to shorten the running time of the program. In p53 data, DysPIA ran significantly faster than others. Using the same personal computer (MacBook Pro, macOS Catalina, i5 2.7 GHz, 8G DDR3 memory), it took about 1.82 s for 1,000 permutations while the others took 23.80 min to 2.03 h (∼800–4,000 times slower). When increasing to 10,000 permutations, the difference became much greater (∼1,700–8,000 times). The details are shown in Figure 4.

The results of DysPIA with fast procedure were also compared to the regular calculation while keeping all the other steps the same. The Pearson (Spearman) correlation coefficient of *P*-values between them was 0.9827 (0.9825) under 1,000 permutations. Therefore, DysPIA with fast calculation kept high consistency with the regular method while it was much faster.

### Number of Permutations

Different numbers of permutations were compared in all the methods based on p53 data. The Pearson (Spearman) correlation coefficients of *P*-values between 1,000 and 10,000 permutations are shown in Table 3. In DysPIA, GSCA, and GSNCA, the correlation coefficients were around 0.998 while they were around 0.90 in ESEA. We further compared the results among 1,000, 10,000, and 100,000 permutations using DysPIA (Supplementary Table 3). Firstly, there was no difference among them if a threshold *P*-value < 0.01 was used. We got exactly the same 17 significant pathways using 1,000, 10,000, or 100,000 permutations. Secondly, 10, 9, and 10 significant pathways were identified if a threshold FDR < 0.05 was used. Nine of these pathways were the same, and pathway “Morphine addiction” can be identified in the 1,000 and 100,000 permutation groups (FDR = 0.0323 and 0.0267), but not in the 10,000 permutation group (FDR = 0.0598, close to the significant level 0.05). Thirdly, some *P*-values and FDRs were significantly different among them due to the precision of different permutations. For example, *P*-values of pathway “Graft-versus-host disease” were 0.0002, 0.0002, and 2.01e-05 (FDRs: 0.0159, 0.0188, and 0.0024) in 1000, 10,000 and 100,000 permutations; *P*-values of pathway “Antigen processing and presentation” were 0.0002, 0.0002, and 2.02e-05 (FDRs: 0.0159, 0.0188, and 0.0024) in 1,000, 10,000, and 100,000 permutations. In these two pathways, the enrichment scores (ES) based on the real data were lower than all the ES based on permutation, and the *P*-values were significantly different among different permutations due to different precisions. These results indicated that the numbers of permutations could affect the *P*-values and FDRs and the 1,000 permutation was not good enough while a large number of pathways were tested. Ten thousand or more permutations were recommended, and it was still very fast in DysPIA. The following results were based on 10,000 permutations.

**TABLE 3:** The correlation coefficients of *P*-value between 1,000 and 10,000 permutations.

Method	PCC	SCC
DysPIA	0.9986	0.9983
ESEA	0.9064	0.9037
GSCA	0.9982	0.9976
GSNCA	0.9986	0.9981

*PCC, Pearson correlation coefficient; SCC, Spearman correlation coefficient.*

### Comparison Between DysPIA and Other Methods

We compared the results of these methods based on p53 data. As shown in Table 4, there were low correlations between these methods. The Pearson (Spearman) correlation coefficients of the *P*-values among ESEA, GSCA, and GSNCA were pretty low (−0.03∼0.05), and the correlation coefficients between DysPIA and ESEA/GSCA were just a little higher (0.10∼0.19).

**TABLE 4:** The correlation of *P*-values between methods.

Method	DysPIA	ESEA	GSCA	GSNCA
DysPIA	1	0.1900	0.1138	−0.1011
ESEA	0.1967	1	0.0516	−0.0308
GSCA	0.1083	0.0531	1	0.0150
GSNCA	−0.1100	−0.0308	0.0222	1

*The upper triangle above the diagonal (number 1) in Table 4 is the Pearson correlation coefficient, and the lower triangle under the diagonal is the Spearman correlation coefficient.*

When the significant threshold was set to *FDR* < 0.05, there were 10 significant dysregulated pathways in DysPIA (the top 10 items in Supplementary Table 4). However, there was no significant pathway in ESEA, GSCA, or GSNCA under *FDR* < 0.05. Then the significant threshold was relaxed to *p* < 0.01; there were 17, 7, 1, and 1 significant dysregulated pathways in DysPIA, ESEA, GSCA, and GSNCA, respectively, and only one in common between DysPIA and ESEA. The detailed results of DysPIA, ESEA, GSCA, and GSNCA are shown in Supplementary Tables 4–7, respectively.

The significant dysregulated pathways were further literature verified. In DysPIA, 16 out of 17 pathways were verified by previous studies (94.1%), while five out of seven pathways in ESEA could be verified (71.4%). The significant dysregulated pathways identified by GSCA and GSNCA were also verified. Specifically, in this p53 mutation study, pathway “p53 signaling pathway” can only be identified in DysPIA. These results showed that DysPIA can mine more dysregulated pathways with a high confidence.

### Dysregulated Pathways in Breast Cancer Relapse

The dysregulated pathways in breast cancer relapse with different ER status were identified based on the dataset GSE2034 using the DysPIA method. Under the threshold of *FDR* < 0.05, there were 14 and 6 significant dysregulated pathways in the “ER+” group and “ER-” group, respectively (Supplementary Tables 8, 9). However, there was only one pathway (antigen processing and presentation) in common, which meant the mechanisms of breast cancer relapse were quite different between the two groups with different ER status. The literature verification was performed on these significant pathways. In the ER+ group, 13 out of 14 pathways (92.9%) had been verified by the existing studies, and 5 out of 6 (83.3%) in the ER- group (Supplementary Tables 8, 9).

### Dysregulated Pathways Between Breast Cancer Subtypes

For each pair of subtypes, the number of significant dysregulated pathways (*FDR* < 0.05 and *FDR* < 0.01) are listed in Table 5. The smaller the number, the more similar between the two subtypes.

**TABLE 5:** Number of dysregulated pathways between subtypes.

Subtype	Basal	Her2	LumA	LumB
Basal	–	53	75	50
Her2	33	–	49	19
LumA	48	20	–	42
LumB	20	10	26	–

*The upper triangle above the diagonal (symbol “-”) in Table 5 is the number when the significance threshold FDR < 0.05, and the lower triangle under the diagonal is the number when FDR < 0.01.*

Subtypes Her2 and LumB were much closer than others, followed by LumA and LumB, while subtype Basal was much different from others. These results were consistent with the previously reports based on PAM50 gene expression (Bastien et al., 2012; Hu et al., 2013; Wallden et al., 2015).

To test the robustness of the proposed method, 80% of the samples in each subtype were randomly selected and used to identify dysregulated pathways by DysPIA. Then, the FDRs were compared to the results using the whole dataset based on correlation analyses. This process was repeated for five times. The average Pearson (Spearman) correlation coefficient was around 0.8, which showed the robustness of DysPIA. The detailed results are shown in Supplementary Tables 10, 11.

## Discussion

Compared to DE and DV, DC/dysregulation analysis considers the dysregulation of two genes between different conditions. Therefore, the DC-based pathway analysis considered the gene regulation relationships while most DE and DV-based methods did not. DC-based pathway analysis can identify some significant dysregulated pathways related to the phonotype interest with different mechanisms to the significant related pathways identified by DE and DV methods.

In DysPIA, the idea of CILP was adopted. There was a score for each sample and gene pair, then a T-test-based statistic was used to represent the gene-pair dysregulation level. In this analysis, the full sample information was used, and sample sizes of different conditions were considered in the calculation. On the other hand, in the PCC-based methods, the PCC was first calculated, and the sample sizes were not considered.

There were regulations or some other relationships between gene pairs in the pathways, which means they were not randomly selected. We compared the absolute dysregulated scores between two groups, the observed gene-pair set from pathways, and the randomly selected gene pairs. The results confirmed that there was a significant difference between them. Therefore, the combined gene-pair background was much sufficient than the observed gene pairs from pathways only. DysPIA was much faster thanks to the fast calculation algorithm for pre-ranked data, and the results confirmed the consistence between the fast method and the classic method.

Unfortunately, there were only a few common pathways identified from different methods. There are several possible reasons. The first was that the gene regulations were not fully considered in GSCA and GSNCA. In their analyses, the test statistic was based on all the possible gene pairs between genes in each pathway. Therefore, the priori regulation knowledge in pathways was not considered but just considered the pathways as gene sets. DysPIA and ESEA considered the existed gene pairs in the pathways in the analysis. The second was that there were different measures in different methods. While PCC or similar measures were used in most methods, the MI-based Edge Score in ESEA was novel but has less directly biological meaning. In p53 mutation data, DysPIA identified more significant dysregulated pathways than other methods under the same threshold with high literature validation rate. The pathway “p53 signaling pathway” can only be identified by DysPIA. In the application of breast cancer relapse dataset and breast cancer subtype dataset, DysPIA also identified several dysregulated pathways which can be verified. Both the simulation and real data results showed that DysPIA is effective and has a great biological significance.

## Conclusion

In DysPIA, the gene regulation information in the pathway is considered, and it provides new insight into pathway analysis area. Both in simulation study and in real datasets, the results show that DysPIA is effective and fast and has a great biological significance. However, there are only a few common pathways identified from different methods. Therefore, using different types of pathway analysis, methods are recommended so that more accurate risk pathways will be identified. The R package “DysPIA” has been constructed and is publicly available on R CRAN at https://cran.r-project.org/web/packages/DysPIA/index.html. Another R dataset package “DysPIAData” containing the latest gene-pair background and pathway list has been publicly available on R CRAN at https://cran.r-project.org/web/packages/DysPIAData/index.html.

## Data Availability Statement

The original contributions presented in the study are included in the article/Supplementary Material, further inquiries can be directed to the corresponding authors.

## Author Contributions

LW, JL, and XL: conceptualization. KL and JL: methodology. LW and WX: software and data curation. WX and ZW: validation. JL: resources. LW, KL, WX, and ZW: writing—original draft preparation. XL, WF, and JL: writing–review and editing. All authors have read and agreed to the published version of the manuscript.

## Conflict of Interest

The authors declare that the research was conducted in the absence of any commercial or financial relationships that could be construed as a potential conflict of interest.

## Footnotes

1 https://cran.r-project.org/web/packages/DysPIA/index.html

2 https://cran.r-project.org/web/packages/DysPIAData/index.html

3 https://github.com/lemonwang2020

## Supplementary Material

Supplementary Table 1 Simulated pathways with a selected sample size in simulation study.

Supplementary Table 2 *P*-values of *t*-test among the absolute DysGPS scores from different backgrounds.

Supplementary Table 3 DysPIA results on p53 data with different backgrounds and permutations.

Supplementary Table 4 Significant dysregulated pathways by DysPIA on p53 data (*p* < 0.01).

Supplementary Table 5 Significant dysregulated pathways by ESEA on p53 data (*p* < 0.01).

Supplementary Table 6 Significant dysregulated pathways by GSCA on p53 data (*p* < 0.01).

Supplementary Table 7 Significant dysregulated pathways by GSNCA on p53 data (*p* < 0.01).

Supplementary Table 8 Dysregulated pathways identified by DysPIA in ER+ group (*FDR* < 0.05).

Supplementary Table 9 Dysregulated pathways identified by DysPIA in ER- group (*FDR* < 0.05).

Supplementary Table 10 The Pearson correlation coefficient of results based on 80% and 100% data.

Supplementary Table 11 The Spearman correlation coefficient of results based on 80% and 100% data.
