# Supplementary material for: DysPIA: A Novel Dysregulated Pathway Identification Analysis Method
Source: Front Genet. 2021 Jul 5;12:647653. doi: 10.3389/fgene.2021.647653 (PMC8287415; doi:10.3389/fgene.2021.647653)
Supplement: Supplementary Table 1 — Simulated pathways with a selected sample size in simulation study. [file Table_3.DOCX]

**Supplementary Table S1.** Simulated pathways with a selected sample size in simulation study

| **Simulation** | **Gene pair type** | **NDP**  **(180)** | **SGCDP**  **(5)** | **WGCDP**  **(5)** | **WLCDP**  **(5)** | **SLCDP**  **(5)** |
| --- | --- | --- | --- | --- | --- | --- |
| Simulation 1 | GCDG | 100% | 20% | 15% | 5% | 0% |
|  | NDG |  | 80% | 80% | 80% | 80% |
|  | LCDG |  | 0% | 5% | 15% | 20% |
| Simulation 2 | GCDG | 100% | 30% | 20% | 10% | 0% |
|  | NDG |  | 70% | 70% | 70% | 70% |
|  | LCDG |  | 0% | 10% | 20% | 30% |
| Simulation 3 | GCDG | 100% | 40% | 25% | 15% | 0% |
|  | NDG |  | 60% | 60% | 60% | 60% |
|  | LCDG |  | 0% | 15% | 25% | 40% |
| Simulation 4 | GCDG | 100% | 50% | 30% | 20% | 0% |
|  | NDG |  | 50% | 50% | 50% | 50% |
|  | LCDG |  | 0% | 20% | 30% | 50% |
| Simulation 5 | GCDG | 100% | 60% | 35% | 25% | 0% |
|  | NDG |  | 40% | 40% | 40% | 40% |
|  | LCDG |  | 0% | 25% | 35% | 60% |

*GCDG: Gain of Correlation Dysregulated Gene pair*

*NDG: Non-Dysregulated Gene pair*

*LCDG: Loss of Correlation Dysregulated Gene pair*

*NDP: Non-Dysregulated pathway*

*SGCDP: Strong Gain of Correlation Dysregulated pathway*

*WGCDP: Week Gain of Correlation Dysregulated pathway*

*WLCDP: Week Loss of Correlation Dysregulated pathway*

*SLCDP: Strong Loss of Correlation Dysregulated pathway*

**Supplementary Table S4.** Significant dysregulated pathways by DysPIA on p53 data (*p*<0.01)

| **No.** | **Pathway** | ***p*-value** | ***FDR*** | **References** |
| --- | --- | --- | --- | --- |
| 1 | Rap1 signaling pathway | 1.79e-4 | 0.0193 | (Boettner and Van Aelst, 2009; Huang, et al., 2018) |
| 2 | Steroid hormone biosynthesis | 2.15e-4 | 0.0193 | (Li, et al., 1996) |
| 3 | Herpes simplex virus 1 infection | 3.79e-4 | 0.0193 | (Boutell and Everett, 2004; Maruzuru, et al., 2013) |
| 4 | Tight junction | 3.82e-4 | 0.0193 | (Kulka, et al., 2009) |
| 5 | Graft-versus-host disease | 4.09e-4 | 0.0193 | (Yada, et al., 2005) |
| 6 | Morphine addiction | 9.39e-4 | 0.0324 | (Tsujikawa, et al., 2009) |
| 7 | Melanoma | 9.65e-4 | 0.0324 | (Houben, et al., 2011; Zerp, et al., 1999) |
| 8 | Regulation of actin cytoskeleton | 0.0011 | 0.0324 | (Croft, et al., 2011) |
| 9 | Metabolism of xenobiotics by cytochrome P450 | 0.0013 | 0.0336 | (Anzenbacher and Anzenbacherova, 2001; Krais, et al., 2016) |
| 10 | AMPK signaling pathway | 0.0021 | 0.0491 | (Zhou, et al., 2014) |
| 11 | **p53 signaling pathway** | 0.0035 | 0.0706 | (Issaeva, 2019) |
| 12 | Glycosphingolipid biosynthesis - lacto and neolacto series | 0.0037 | 0.0706 | (Liu, et al., 2013) |
| 13 | Phenylalanine metabolism | 0.0039 | 0.0706 |  |
| 14 | Phosphatidylinositol signaling system | 0.0044 | 0.0747 | (Naguib, et al., 2015) |
| 15 | Glutathione metabolism | 0.0054 | 0.0851 | (Lacroix, et al., 2020) |
| 16 | PD-L1 expression and PD-1 checkpoint pathway in cancer | 0.0060 | 0.0883 | (Xu and Zhang, 2018) |
| 17 | One carbon pool by folate | 0.0065 | 0.0896 | (Curtin, et al., 2011) |

**Supplementary Table S5.** Significant dysregulated pathways by ESEA on p53 data (*p*<0.01)

| **No.** | **Pathway** | ***p*-value** | ***FDR*** | **References** |
| --- | --- | --- | --- | --- |
| 1 | Cysteine and methionine metabolism | 0.0015 | 0.0649 | (Gao, et al., 2019) |
| 2 | Cholinergic synapse | 0.0017 | 0.0649 |  |
| 3 | Longevity regulating pathway - multiple species | 0.0018 | 0.0649 | (Donehower, 2009) |
| 4 | Legionellosis | 0.002 | 0.0649 | (Herkt, et al., 2020) |
| 5 | ECM-receptor interaction | 0.0022 | 0.0649 | (Ilic, et al., 1998) |
| 6 | Glutamatergic synapse | 0.0028 | 0.0734 |  |
| 7 | Rap1 signaling pathway | 0.0058 | 0.1369 | (Huang, et al., 2018) |

**Supplementary Table S6.** Significant dysregulated pathways by GSCA on p53 data (*p*<0.01)

| **No.** | **Pathway** | ***p*-value** | ***FDR*** | **References** |
| --- | --- | --- | --- | --- |
| 1 | Fatty acid biosynthesis | 0.0033 | 0.8541 | (Parrales and Iwakuma, 2016) |

**Supplementary Table S7.** Significant dysregulated pathways by GSNCA on p53 data (*p*<0.01)

| **No.** | **Pathway** | ***p*-value** | ***FDR*** | | **References** |
| --- | --- | --- | --- | --- | --- |
| 1 | Autophagy - other | 0.0021 | | 0.6551 | (Mrakovcic and Frohlich, 2018) |

**Supplementary Table S8.** Dysregulated pathways identified by DysPIA in ER+ group *(FDR*<0.05)

| **No.** | **Pathway** | ***p*-value** | ***FDR*** | **References** |
| --- | --- | --- | --- | --- |
| 1 | Osteoclast differentiation | 1.42e-4 | 0.0068 | (Sawant, et al., 2013; Schramek, et al., 2010) |
| 2 | Cytokine-cytokine receptor interaction | 1.44e-4 | 0.0068 | (Van der Auwera, et al., 2010; Zhang, et al., 2017) |
| 3 | Natural killer cell mediated cytotoxicity | 1.47e-4 | 0.0068 | (Ames, et al., 2009; Shen, et al., 2017) |
| 4 | Antigen processing and presentation | 1.57e-4 | 0.0068 | (Liu, et al., 2012; Scanlan and Jager, 2001) |
| 5 | Graft-versus-host disease | 1.71e-4 | 0.0068 | (Eibl, et al., 1996; Holmberg, et al., 2006) |
| 6 | Allograft rejection | 1.78e-4 | 0.0068 |  |
| 7 | Ascorbate and aldarate metabolism | 1.83e-4 | 0.0068 | (Engel, et al., 2012) |
| 8 | NOD-like receptor signaling pathway | 2.96e-4 | 0.0096 | (Peng, et al., 2016) |
| 9 | Cellular senescence | 4.23e-4 | 0.0122 | (Angelini, et al., 2013; Gao, et al., 2016) |
| 10 | Drug metabolism - other enzymes | 5.41e-4 | 0.0140 | (Wegman, et al., 2005) |
| 11 | RIG-I-like receptor signaling pathway | 6.35e-4 | 0.0149 | (Ranoa, et al., 2016) |
| 12 | Prolactin signaling pathway | 0.0015 | 0.0334 | (Aksamitiene, et al., 2011; Barcus, et al., 2013) |
| 13 | Measles | 0.0018 | 0.0353 | (McDonald, et al., 2006; Sugiyama, et al., 2013) |
| 14 | Tyrosine metabolism | 0.0021 | 0.0381 | (Polli, et al., 2009) |

**Supplementary Table S9.** Dysregulated pathways identified by DysPIA in ER- group (*FDR*<0.05)

| **No.** | **Pathway** | ***p*-value** | ***FDR*** | **References** |
| --- | --- | --- | --- | --- |
| 1 | Retinol metabolism | 1.75e-4 | 0.0152 | (Chen, et al., 1997; Hayden and Satre, 2002) |
| 2 | Antigen processing and presentation | 1.84e-4 | 0.0152 | (Liu, et al., 2012; Scanlan and Jager, 2001) |
| 3 | Human T-cell leukemia virus 1 infection | 2.26e-4 | 0.0152 |  |
| 4 | Herpes simplex virus 1 infection | 2.34e-4 | 0.0151 | (Chen, et al., 2016; Toda, et al., 1998) |
| 5 | Metabolism of xenobiotics by cytochrome P450 | 3.53e-4 | 0.0183 | (Li, et al., 2017; Murray, et al., 1993) |

**Supplementary Table S10.** The Pearson correlation coefficient of results based on 80% and 100% data

|  | **Pearson correlation coefficient** | | | | | |
| --- | --- | --- | --- | --- | --- | --- |
| **Subtype pair** | **round1** | **round2** | **round3** | **round4** | **round5** | **Average** |
| Basal_Her2 | 0.7732 | 0.7088 | 0.7629 | 0.7647 | 0.6337 | **0.7287** |
| Basal_LumA | 0.8681 | 0.8802 | 0.8226 | 0.8842 | 0.8466 | **0.8603** |
| Basal_LumB | 0.8265 | 0.7971 | 0.8368 | 0.8225 | 0.7923 | **0.8150** |
| Her2_LumA | 0.8052 | 0.7498 | 0.8182 | 0.7142 | 0.6551 | **0.7485** |
| Her2_LumB | 0.8302 | 0.7470 | 0.7025 | 0.7391 | 0.7278 | **0.7493** |
| LumA_LumB | 0.8357 | 0.7958 | 0.7255 | 0.8273 | 0.8279 | **0.8024** |

**Supplementary Table S11.** The Spearman correlation coefficient of results based on 80% and 100% data

|  | **Spearman correlation coefficient** | | | | | |
| --- | --- | --- | --- | --- | --- | --- |
| **Subtype pair** | **round1** | **round2** | **round3** | **round4** | **round5** | **Average** |
| Basal_Her2 | 0.8110 | 0.7525 | 0.7932 | 0.7910 | 0.6875 | **0.7670** |
| Basal_LumA | 0.9006 | 0.9162 | 0.8704 | 0.9197 | 0.8824 | **0.8978** |
| Basal_LumB | 0.8386 | 0.8133 | 0.8424 | 0.8147 | 0.8021 | **0.8222** |
| Her2_LumA | 0.8228 | 0.7693 | 0.8323 | 0.7668 | 0.7197 | **0.7822** |
| Her2_LumB | 0.8028 | 0.6953 | 0.6368 | 0.7054 | 0.6959 | **0.7073** |
| LumA_LumB | 0.8505 | 0.8034 | 0.7342 | 0.8464 | 0.8329 | **0.8135** |

References

Aksamitiene, E.*, et al.* Prolactin-stimulated activation of ERK1/2 mitogen-activated protein kinases is controlled by PI3-kinase/Rac/PAK signaling pathway in breast cancer cells. *Cell Signal* 2011;23(11):1794-1805.

Ames, E., Hallett, W.H. and Murphy, W.J. Sensitization of human breast cancer cells to natural killer cell-mediated cytotoxicity by proteasome inhibition. *Clin Exp Immunol* 2009;155(3):504-513.

Angelini, P.D.*, et al.* Constitutive HER2 signaling promotes breast cancer metastasis through cellular senescence. *Cancer Res* 2013;73(1):450-458.

Anzenbacher, P. and Anzenbacherova, E. Cytochromes P450 and metabolism of xenobiotics. *Cell Mol Life Sci* 2001;58(5-6):737-747.

Barcus, C.E.*, et al.* Stiff collagen matrices increase tumorigenic prolactin signaling in breast cancer cells. *J Biol Chem* 2013;288(18):12722-12732.

Boettner, B. and Van Aelst, L. Control of cell adhesion dynamics by Rap1 signaling. *Curr Opin Cell Biol* 2009;21(5):684-693.

Boutell, C. and Everett, R.D. Herpes simplex virus type 1 infection induces the stabilization of p53 in a USP7- and ATM-independent manner. *J Virol* 2004;78(15):8068-8077.

Chen, A.C.*, et al.* Human breast cancer cells and normal mammary epithelial cells: retinol metabolism and growth inhibition by the retinol metabolite 4-oxoretinol. *Cancer Res* 1997;57(20):4642-4651.

Chen, X.*, et al.* A combinational therapy of EGFR-CAR NK cells and oncolytic herpes simplex virus 1 for breast cancer brain metastases. *Oncotarget* 2016;7(19):27764-27777.

Croft, D.R.*, et al.* p53-mediated transcriptional regulation and activation of the actin cytoskeleton regulatory RhoC to LIMK2 signaling pathway promotes cell survival. *Cell Res* 2011;21(4):666-682.

Curtin, K.*, et al.* Nutrients in folate-mediated, one-carbon metabolism and the risk of rectal tumors in men and women. *Nutr Cancer* 2011;63(3):357-366.

Donehower, L.A. Longevity regulation in flies: a role for p53. *Aging (Albany NY)* 2009;1(1):6-8.

Eibl, B.*, et al.* Evidence for a graft-versus-tumor effect in a patient treated with marrow ablative chemotherapy and allogeneic bone marrow transplantation for breast cancer. *Blood* 1996;88(4):1501-1508.

Engel, N.*, et al.* Metabolic profiling reveals sphingosine-1-phosphate kinase 2 and lyase as key targets of (phyto-) estrogen action in the breast cancer cell line MCF-7 and not in MCF-12A. *PLoS One* 2012;7(10):e47833.

Gao, X.*, et al.* Dietary methionine influences therapy in mouse cancer models and alters human metabolism. *Nature* 2019;572(7769):397-401.

Gao, Y.*, et al.* The dual function of PRMT1 in modulating epithelial-mesenchymal transition and cellular senescence in breast cancer cells through regulation of ZEB1. *Sci Rep* 2016;6:19874.

Hayden, L.J. and Satre, M.A. Alterations in cellular retinol metabolism contribute to differential retinoid responsiveness in normal human mammary epithelial cells versus breast cancer cells. *Breast Cancer Res Treat* 2002;72(2):95-105.

Herkt, C.E.*, et al.* A MicroRNA Network Controls Legionella pneumophila Replication in Human Macrophages via LGALS8 and MX1. *mBio* 2020;11(2).

Holmberg, L.*, et al.* Gastrointestinal graft-versus-host disease in recipients of autologous hematopoietic stem cells: incidence, risk factors, and outcome. *Biol Blood Marrow Transplant* 2006;12(2):226-234.

Houben, R.*, et al.* High-level expression of wild-type p53 in melanoma cells is frequently associated with inactivity in p53 reporter gene assays. *PLoS One* 2011;6(7):e22096.

Huang, R., Liao, X. and Li, Q. Identification of key pathways and genes in TP53 mutation acute myeloid leukemia: evidence from bioinformatics analysis. *Onco Targets Ther* 2018;11:163-173.

Ilic, D.*, et al.* Extracellular matrix survival signals transduced by focal adhesion kinase suppress p53-mediated apoptosis. *J Cell Biol* 1998;143(2):547-560.

Issaeva, N. p53 Signaling in Cancers. *Cancers (Basel)* 2019;11(3).

Krais, A.M.*, et al.* The impact of p53 on DNA damage and metabolic activation of the environmental carcinogen benzo[a]pyrene: effects in Trp53(+/+), Trp53(+/-) and Trp53(-/-) mice. *Arch Toxicol* 2016;90(4):839-851.

Kulka, J.*, et al.* Expression of tight junction protein claudin-4 in basal-like breast carcinomas. *Pathol Oncol Res* 2009;15(1):59-64.

Lacroix, M.*, et al.* Metabolic functions of the tumor suppressor p53: Implications in normal physiology, metabolic disorders, and cancer. *Mol Metab* 2020;33:2-22.

Li, S.F.*, et al.* Stepwise abnormality of sex steroid hormone receptors, tumor suppressor gene products (p53 and Rb), and cyclin E in uterine endometrioid carcinoma. *Cancer* 1996;77(2):321-329.

Li, Y.*, et al.* Tumoral expression of drug and xenobiotic metabolizing enzymes in breast cancer patients of different ethnicities with implications to personalized medicine. *Sci Rep* 2017;7(1):4747.

Liu, Y.*, et al.* Expression of antigen processing and presenting molecules in brain metastasis of breast cancer. *Cancer Immunol Immunother* 2012;61(6):789-801.

Liu, Y.Y., Hill, R.A. and Li, Y.T. Ceramide glycosylation catalyzed by glucosylceramide synthase and cancer drug resistance. *Adv Cancer Res* 2013;117:59-89.

Maruzuru, Y.*, et al.* Roles of p53 in herpes simplex virus 1 replication. *J Virol* 2013;87(16):9323-9332.

McDonald, C.J.*, et al.* A measles virus vaccine strain derivative as a novel oncolytic agent against breast cancer. *Breast Cancer Res Treat* 2006;99(2):177-184.

Mrakovcic, M. and Frohlich, L.F. p53-Mediated Molecular Control of Autophagy in Tumor Cells. *Biomolecules* 2018;8(2).

Murray, G.I.*, et al.* Expression of xenobiotic metabolizing enzymes in breast cancer. *J Pathol* 1993;169(3):347-353.

Naguib, A.*, et al.* p53 mutations change phosphatidylinositol acyl chain composition. *Cell Rep* 2015;10(1):8-19.

Parrales, A. and Iwakuma, T. p53 as a Regulator of Lipid Metabolism in Cancer. *Int J Mol Sci* 2016;17(12).

Peng, L.*, et al.* Ubiquitin specific protease 21 upregulation in breast cancer promotes cell tumorigenic capability and is associated with the NOD-like receptor signaling pathway. *Oncol Lett* 2016;12(6):4531-4537.

Polli, J.W.*, et al.* An unexpected synergist role of P-glycoprotein and breast cancer resistance protein on the central nervous system penetration of the tyrosine kinase inhibitor lapatinib (N-{3-chloro-4-[(3-fluorobenzyl)oxy]phenyl}-6-[5-({[2-(methylsulfonyl)ethyl]amino }methyl)-2-furyl]-4-quinazolinamine; GW572016). *Drug Metab Dispos* 2009;37(2):439-442.

Ranoa, D.R.*, et al.* Cancer therapies activate RIG-I-like receptor pathway through endogenous non-coding RNAs. *Oncotarget* 2016;7(18):26496-26515.

Sawant, A.*, et al.* Myeloid-derived suppressor cells function as novel osteoclast progenitors enhancing bone loss in breast cancer. *Cancer Res* 2013;73(2):672-682.

Scanlan, M.J. and Jager, D. Challenges to the development of antigen-specific breast cancer vaccines. *Breast Cancer Res* 2001;3(2):95-98.

Schramek, D.*, et al.* Osteoclast differentiation factor RANKL controls development of progestin-driven mammary cancer. *Nature* 2010;468(7320):98-102.

Shen, J.*, et al.* Silencing NKG2D ligand-targeting miRNAs enhances natural killer cell-mediated cytotoxicity in breast cancer. *Cell Death Dis* 2017;8(4):e2740.

Sugiyama, T.*, et al.* Measles virus selectively blind to signaling lymphocyte activation molecule as a novel oncolytic virus for breast cancer treatment. *Gene Ther* 2013;20(3):338-347.

Toda, M., Rabkin, S.D. and Martuza, R.L. Treatment of human breast cancer in a brain metastatic model by G207, a replication-competent multimutated herpes simplex virus 1. *Hum Gene Ther* 1998;9(15):2177-2185.

Tsujikawa, H.*, et al.* Morphine induces DNA damage and P53 activation in CD3+ T cells. *Biochim Biophys Acta* 2009;1790(8):793-799.

Van der Auwera, I.*, et al.* Array-based DNA methylation profiling for breast cancer subtype discrimination. *PLoS One* 2010;5(9):e12616.

Wegman, P.*, et al.* Genotype of metabolic enzymes and the benefit of tamoxifen in postmenopausal breast cancer patients. *Breast Cancer Res* 2005;7(3):R284-290.

Xu, C. and Zhang, Z.H. Correlation between Programmed Death-1 Ligand-1 and p53 in Patients with Lung Adenocarcinoma. *Chin Med J (Engl)* 2018;131(8):990-993.

Yada, S.*, et al.* The role of p53 and Fas in a model of acute murine graft-versus-host disease. *J Immunol* 2005;174(3):1291-1297.

Zerp, S.F.*, et al.* p53 mutations in human cutaneous melanoma correlate with sun exposure but are not always involved in melanomagenesis. *Br J Cancer* 1999;79(5-6):921-926.

Zhang, L.*, et al.* Identification and characterization of biomarkers and their functions for Lapatinib-resistant breast cancer. *Med Oncol* 2017;34(5):89.

Zhou, G.*, et al.* Gain-of-function mutant p53 promotes cell growth and cancer cell metabolism via inhibition of AMPK activation. *Mol Cell* 2014;54(6):960-974.
